# Perfluorooctane Sulfonate (PFOS) and Related Perfluorinated Compounds in Human Maternal and Cord Blood Samples: Assessment of PFOS Exposure in a Susceptible Population during Pregnancy

**DOI:** 10.1289/ehp.6864

**Published:** 2004-04-13

**Authors:** Koichi Inoue, Fumio Okada, Rie Ito, Shizue Kato, Seiko Sasaki, Sonomi Nakajima, Akiko Uno, Yasuaki Saijo, Fumihiro Sata, Yoshihiro Yoshimura, Reiko Kishi, Hiroyuki Nakazawa

**Affiliations:** ^1^Department of Analytical Chemistry, Faculty of Pharmaceutical Sciences, Hoshi University, Tokyo, Japan; ^2^Department of Public Health, Hokkaido University Graduate School of Medicine, Hokkaido, Japan

**Keywords:** cord blood, fluorinated organic compounds, human, PFOA, PFOS, PFOSA, pregnancy

## Abstract

Fluorinated organic compounds (FOCs), such as perfluorooctane sulfonate (PFOS), perfluorooctanoate (PFOA), and perfluorooctane sulfonylamide (PFOSA), are widely used in the manufacture of plastic, electronics, textile, and construction material in the apparel, leather, and upholstery industries. FOCs have been detected in human blood samples. Studies have indicated that FOCs may be detrimental to rodent development possibly by affecting thyroid hormone levels. In the present study, we determined the concentrations of FOCs in maternal and cord blood samples. Pregnant women 17–37 years of age were enrolled as subjects. FOCs in 15 pairs of maternal and cord blood samples were analyzed by liquid chromatography–electrospray mass spectrometry coupled with online extraction. The limits of quantification of PFOS, PFOA, and PFOSA in human plasma or serum were 0.5, 0.5, and 1.0 ng/mL, respectively. The method enables the precise determination of FOCs and can be applied to the detection of FOCs in human blood samples for monitoring human exposure. PFOS concentrations in maternal samples ranged from 4.9 to 17.6 ng/mL, whereas those in fetal samples ranged from 1.6 to 5.3 ng/mL. In contrast, PFOSA was not detected in fetal or maternal samples, whereas PFOA was detected only in maternal samples (range, < 0.5 to 2.3 ng/mL, 4 of 15). Our results revealed a high correlation between PFOS concentrations in maternal and cord blood (*r*^2^ = 0.876). However, we did not find any significant correlations between PFOS concentration in maternal and cord blood samples and age bracket, birth weight, or levels of thyroid-stimulating hormone or free thyroxine. Our study revealed that human fetuses in Japan may be exposed to relatively high levels of FOCs. Further investigation is required to determine the postnatal effects of fetal exposure to FOCs.

Fluorinated organic compounds (FOCs), such as perfluorooctane sulfonate (PFOS), perfluorooctanoate (PFOA), and perfluorooctane sulfonylamide (PFOSA), are stable chemicals with a wide range of industrial and consumer applications (Renner 2001). Recent reports have indicated the presence of these environmental contaminants in wildlife and river water (Giesy and Kannan 2001; Kannan et al. 2001; Saito et al. 2003; Taniyasu et al. 2003). FOCs have been manufactured for > 50 years and are used as refrigerants, surfactants, and polymers and as components of paper coatings, fire retardants, adhesives, cosmetics, and insecticides (Key et al. 1997).

In the United States, PFOS is a stable FOC with many industrial applications. The amount of PFOS used in consumer application totals 5.6 million lb [U.S. Environmental Protection Agency (EPA) 2000]. These findings prompted the major manufacturer of PFOS in the United States, 3M Company, to halt production in the end of 2002 (Renner 2001). However, PFOS is still available on the Japanese market. A recent evaluation of PFOS toxicity in green algae revealed that the no observable effect concentration (NOEC) ranges from 5.3 to 8.2 mg/L (Boudreau et al. 2003). In addition, PFOS is well absorbed but poorly metabolized and excreted, and it has a long half-life (200 days) in monkeys (Seacat et al. 2002). On the other hand, the potential toxicity of PFOS in general is not well characterized, and even less known are the mechanisms underlying its toxic effects. Hepatic toxicity and altered thyroid hormone levels have been found in monkeys and rodents (Luebker et al. 2002; Seacat et al. 2002, 2003). PFOS interferes with mitochondrial bioenergetics, and cell–cell communication has been implicated as a potential mechanism of toxicity (Hu et al. 2002, 2003). Recently, the neuroendocrine effects of PFOS were reported in the rat (Austin et al. 2003). In addition, maternal, developmental, and postnatal toxicities of PFOS were reported in the rat and mouse (Lau et al. 2003; Thibodeaux et al. 2003), showing that exposure to PFOS of pregnant rats and mice leads to significant physiologic alterations and that additional investigations are required to elucidate the pathophysiologic mechanisms.

PFOA is widely used in the production of chemicals and in aircraft and electronic products manufacturing. PFOA does not appear to be biomagnified in animals. However, it is a persistent pollutant, and low concentrations are detected in human blood, according to a risk assessment study (Renner 2003). In the same manner as PFOA, risk assessment of PFOSA is required for evaluating exposure levels and effects.

Because of the ubiquity of FOCs and their potential role in increasing the risk of neuroendocrine and reproductive dysfunction, human exposure assessment studies are urgently needed. A quantitative analysis of four FOCs in 65 human serum samples collected from several biologic supply companies in the United States detected PFOS (6.7–81.5 ng/mL), PFOA [limit of quantitation (LOQ), 35.2 ng/mL], and PFOSA (LOQ, 2.2 ng/mL) (Hansen et al. 2001). A study of human exposure to FOCs found that mean serum PFOS concentration was 17.7 ng/mL in 24 donors (Olsen et al. 2003b). In Japan, there is only one report of exposure in humans (Taniyasu et al. 2003), indicating that PFOS concentrations range from 2.4 to 14 ng/mL. Recently, a rapid and sensitive method for measuring 15 FOCs in human breast milk and serum has been developed (Calafat et al. 2003). The study of human exposure to FOCs requires information about the concentration of these toxicants in a non-occupationally exposed population. However, to our knowledge, there is no study of FOCs in human maternal and cord blood samples for fetal risk assessment. Human fetal and maternal exposure assessment studies are urgently needed because maternal and developmental toxicities of PFOS have been indicated (Lau et al. 2003; Thibodeaux et al. 2003).

The aim of the present study was to determine human cord and maternal serum concentrations of PFOS, PFOA, and PFOSA for fetal risk assessment. Although based on only a small sample set, our findings indicate the levels of FOCs to which Japanese women are exposed. We developed an easy, reliable, and high-throughput analytical method that uses liquid chromatography–electrospray mass spectrometry (LC-MS) coupled with online extraction to measure specific FOCs in human plasma and serum samples. This method enables the precise determination of standards in human blood samples and can be applied to the detection of PFOS, PFOA, and PFOSA in human blood samples for monitoring human exposure.

## Materials and Methods

### Clinical materials.

We recruited subjects between February and July 2003 at Sapporo Toho Hospitals in Hokkaido, Japan. This study was conducted with all the subjects’ written informed consent and was approved by the institutional ethical board for epidemiologic studies at the Hokkaido University Graduate School of Medicine. The data of physical and biologic examinations, laboratory tests, and questionnaires were recorded by the Department of Public Heath, Hokkaido University Graduate School of Medicine. Blood was sampled from pregnant women (*n* = 15; Table 1) between gestation weeks 38 and 41 (mean ± SD, 39.7 ± 1.05). Cord blood samples were collected immediately after birth by using standard procedure, which included careful cleansing of the cord and strict puncture of the umbilical vein to avoid maternal contamination. Body mass index (BMI) was calculated from the information given in Table 1 (height, prepregnancy weight, and weight at delivery).

### FOC analysis.

PFOS [molecular weight (MW), 538.23; 98% purity], PFOA (MW, 414.07; > 90%), and PFOSA (MW, 199.14; 97%) were purchased from Wako Pure Chemical Inc. (Osaka, Japan), Fluka Chemie AG (Buchs, Switzerland), and ABCR GmbH & Co. (Im Schlehert, Germany). The internal standard (perfluorodecanoic acid) was purchased from Lancaster Company, Inc. (Morecambe, UK). Other reagents and solvents were of HPLC grade and were purchased from Wako Pure Chemical Inc. (Osaka, Japan). The distilled water purification system was Milli-Q gradient A 10 with an EDS polisher (Millipore, Bedford, MA, USA).

LC-MS with electrospray ionization was performed using an Agilent 1100 MSD-SL system (Agilent Technologies, Palo Alto, CA, USA). The working conditions were as follows: The drying nitrogen gas temperature was set at 350°C and was introduced into the capillary region at a flow rate of 12 L/min; the capillary was held at a potential of 3,500 V relative to the counter electrode in the negative-ion mode for all compounds. The fragmenter voltages were 220 V for PFOS, 130 V for PFOA, and 170 V for PFOSA during the chromatographic run. The direct injection volume was 30 μL. The column used was Inertsil C_8–3_ (2.1 × 100 mm, 5 μm; GL Sciences Inc., Tokyo, Japan) with a Mightysil RP-18 GP precolumn (2.0 × 5 mm, 5 μm; Kanto Chemical Inc., Osaka, Japan).

The column-switching LC-MS coupled with an on-line extraction system consisted of this LC-MS combined with an LC pump (Shimadzu LC-10 ADvp pump; Shimadzu, Kyoto, Japan) and Oasis HLB extraction column (20 × 2.1 mm, 25 μm; Waters Co., Milford, MA, USA). After a blood sample was injected by an autosampler, it was loaded onto the extraction column by flowing water/methanol (90/10, vol/vol) at a rate of 1.0 mL/min using a Shimadzu pump for 5 min. After on-line extraction for 5 min, the position of the switching valve was changed. This configuration connected the back-flashing extraction column to the analytical column and the MS detector in the flow path of the Agilent pump. After 20 min, the switching valve was returned to its original position. The run time for the assay of the sample mixture was 30 min. Gradient mobile phase of 1.0 mM ammonium acetate in water/acetonitrile (vol/vol) was used at a flow rate of 0.2 mL/min (5–15 min using a linear increase from 65 to 85% acetonitrile solution and holding at 85%).

In the quantitative procedure, standard solutions of PFOS, PFOA, and PFOSA were prepared in aqueous solution to cover the calibration range. Quantitative analysis was performed in the single ion monitoring mode to maximize sensitivity. PFOS, PFOA, and PFOSA concentrations in each sample were calculated relative to the internal standard added to the sample before direct analysis. Calibration curves of PFOS, PFOA, and PFOSA were performed daily for all samples with internal standard. We added 0.3 mL of sample to 0.3 mL of internal standard solution. The mixed sample was centrifuged at 3,000 × *g* for 10 min. The top clear layer was removed to the glass tube. This sample solution was filtered. This solution was analyzed by column-switching LC-MS. We analyzed quality-control materials (spiked samples in 25 ng/mL of PFOS) with each batch of samples on separated days. In the result, this material did not deviate from the 99% confidence interval (CI) (in this case, 99% CI, 24.17–25.53).

The method was developed previously. The compounds were separated by reverse-phase LC with a C_8_ column and detected by MS in the selected ion monitoring mode. When working in the selected ion monitoring mode, the *m/z* ions for PFOS, PFOA, and PFOSA were [M–K]^−^ 499, [M–COOH]^−^ 369, and [M–H]^−^ 498. In addition, the *m/z* ion of the internal standard was designated as [M–COOH]^−^ 469 in the negative ion mode.

The analysis of trace levels of PFOS, PFOA, and PFOSA in biologic samples is complicated by contamination, particularly by leaching from Teflon plastic. Thus, care must be taken to control contamination during experiments and, where possible, to eliminate the contamination. Investigations of PFOS, PFOA, and PFOSA contamination of the Milli-Q water system, the plastic tube, and the LC system produced negative results (below the limit of detection). We investigated whether the recoveries of PFOS, PFOA, and PFOSA (10 and 100 ng/mL) in the samples could be determined by this method. The average recoveries of PFOS, PFOA, and PFOSA ranged from 82.2 to 98.7%, with relative standard deviation < 5.2%. We used this method to assess FOC levels in human blood samples to obtain a reference range.

### Thyroid hormone estimation.

Thyroid-stimulating hormone (TSH) and free thyroxine (T_4_) levels of newborns were measured in Sapporo City Institute of Public Health. Blood specimens on filter paper were collected from infants between 4 and 7 days of age. TSH and free T_4_ were determined in single 0.3-cm disks punched from the same filter-paper blood. TSH and free T_4_ were measured using enzyme-linked immunosorbent assay (TSH: Enzaplate N-TSH, Bayer Co., Tokyo, Japan; free T_4_: Enzaplate N-FT4, Bayer). Detection limits were for TSH, 0.5 μIU/mL, and for free T_4_, 0.20 ng/dL, respectively.

## Results

We were able to realize a low LOQ and rapid analysis using the developed column-switching LC-MS coupled with on-line extraction method. The calculated LOQs when the signal-to-noise ratio was 10 were 0.5 for PFOS, 0.5 for PFOA, and 1.0 ng/mL for PFOSA in human serum samples.

**Figure 1:**
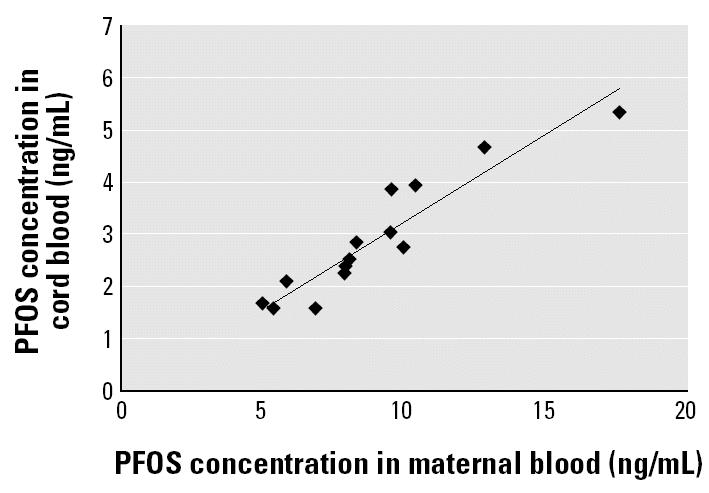
PFOS concentrations in maternal and cord blood samples (*r*^2^ = 0.8759; *y* = 0.3332*x*–0.0877).

**Figure 2:**
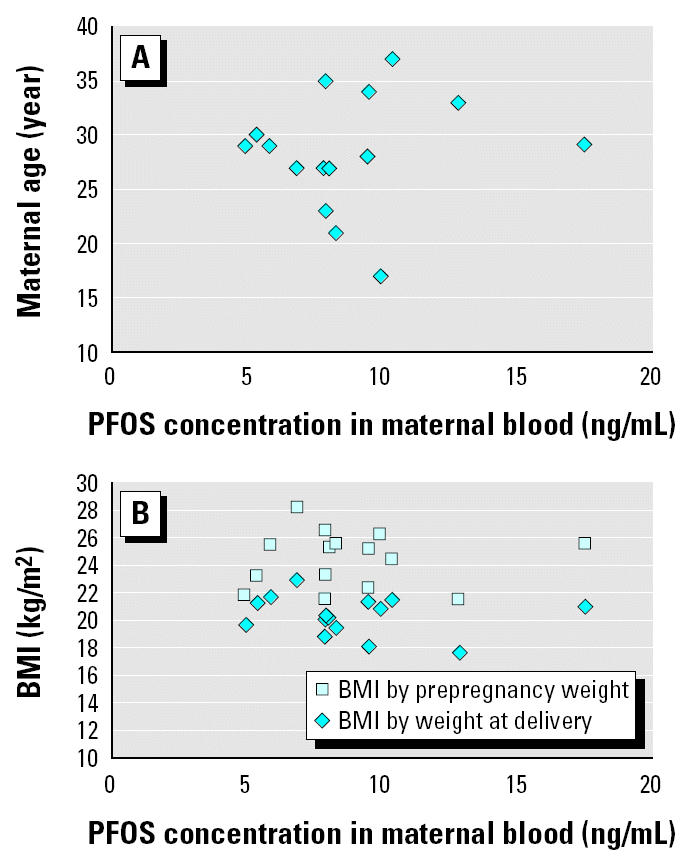
Maternal age (*A*) and BMI (*B*) plotted against PFOS concentration in maternal blood samples (*n* = 15).

**Figure 3:**
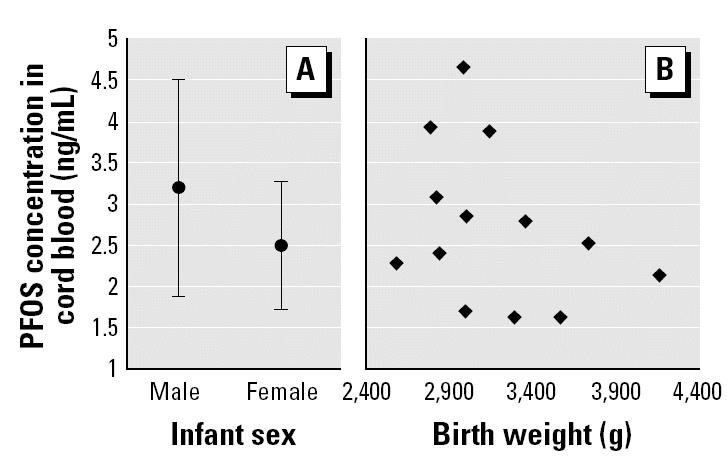
Infants’ sex (*A*) and birth weight (*B*) plotted against PFOS concentration in cord blood samples (*n* = 15). Error bars indicate mean ± SD.

**Figure 4:**
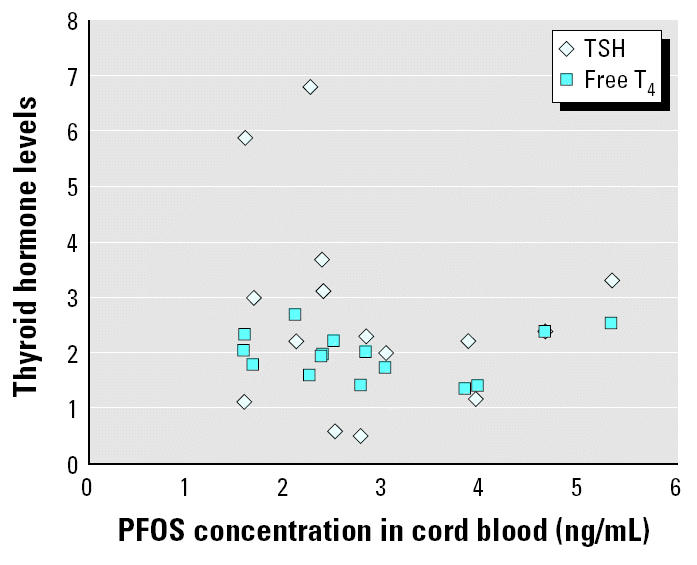
Infants’ thyroid hormones levels (TSH and free T_4_) plotted against PFOS concentration in cord blood samples (*n* = 15).

We analyzed 15 pairs of maternal and cord serum samples for PFOS, PFOA, and PFOSA by this method. In the results (Table 2), the percentage detection of PFOS, PFOA, and PFOSA in the maternal and cord samples was 100% (30 of 30), 10% (3 of 30), and 0% (0 of 30), respectively. The concentrations of PFOS in maternal serum samples ranged from 4.9 to 17.6 ng/mL, whereas those in cord samples ranged from 1.6 to 5.3 ng/mL. The PFOS concentrations in maternal and cord blood samples were highly correlated (*r*^2^ = 0.876) (Figure 1). The average maternal age was 28.4 years, with a range of 17–37 years. In addition, the average BMIs based on prepregnancy weight and on weight at delivery were 20.3 and 24.4, respectively. PFOS concentration was not correlated with these parameters (Figure 2). On the other hand, Figure 3 shows no apparent correlation between cord PFOS concentration and sex and weight at birth. In addition, Figure 4 shows no apparent correlation between cord PFOS concentration and thyroid hormone factors such as TSH and free T_4_.

## Discussion

To the best of our knowledge, this is the first report of PFOS and its related compounds in pregnant Japanese women and fetal cord blood. The issue of the bioavailability of PFOS in human, particularly in pregnant women and their cord blood, is controversial. A study of female human exposure levels of PFOS concentration found that levels in the United States are approximately twice those in Japan (Olsen et al. 2003b; Taniyasu et al. 2003). In contrast, PFOS concentrations in cord blood samples have not been reported so far. Therefore, we cannot compare our cord blood PFOS exposure levels with other data. However, a study of exposure to PFOS during pregnancy in the rat and mouse indicated that the amount of accumulated PFOS is proportional to the treatment dosage and the levels detected in fetal liver; in terms of concentration, the fetal liver level appears to contain approximately half as much PFOS as its maternal counterpart (Thibodeaux et al. 2003). Based on our study, the mean ratio of PFOS concentration in maternal blood to that in cord blood is 0.32 (range, 0.23–0.41). The studies of PFOS exposure during pregnancy in the rat and mouse (Lau et al. 2003; Thibodeaux et al. 2003) support our findings that PFOS accumulation can be measured in humans and that there is a high correlation between PFOS concentrations in maternal and cord blood.

On the other hand, PFOA was detected in a small number of maternal samples but not in cord samples (< 0.1 ng/mL). PFOA may pose a developmental risk to children at concentrations already found in the blood of women and children, according to a U.S. EPA preliminary risk assessment released in April 2003 (Renner 2003). Kannan et al. (2002a) measured FOCs, including PFOA, in bird livers collected from Japan and South Korea and found that the highest concentration of PFOA in the samples was 21 ng/g wet weight. PFOA has been found in environmental samples (Giesy and Kannan 2001; Kannan et al. 2002b). However, we do not know how people are exposed to PFOA according to a nonoccupational exposure assessment study. The occupational exposure levels of PFOA in humans are 1,780 ng/mL with a range of 40–10,060 ng/mL (Olsen et al. 2003a), and 899 ng/mL with a range of 722–1,120 ng/mL (Olsen et al. 2003c). By contrast, nonoccupational exposure to PFOA was found to be at trace levels (range of < 0.5–4.1 ng/mL, 15 of 21) in our other study (Okada et al. 2003). Therefore, we surmise that fetal exposure to PFOA is at trace levels. Recently, the dissociation constants for PFOA binding to human serum albumin (HSA) and the number of PFOA binding sites on HSA were determined (Han et al. 2003). At the same time, Han et al. (2003) predicted that PFOA bound to maternal blood protein may not be able to cross the placental barrier. The reasons for the trace levels of PFOA in fetal blood samples are nonoccupational exposure and the binding of PFOA to maternal blood protein.

Like polychlorinated biphenyls, organochlorine pesticides, and polybrominated diphenyl ethers, PFOS may be able to cross the placental barrier to enter fetal circulation (Covaci et al. 2002; DeKoning and Karmaus 2000; Mazdai et al. 2003; Sala et al. 2001; Waliszewski et al. 2000). Our data suggest that the slope is approximately 0.33 (Figure 1), indicating that PFOS does not pass into the fetal circulation completely; that is, there does seem to be a barrier effect. In contrast to PFOS, however, PFOA and PFOSA cannot cross the placental barrier to enter fetal circulation. PFOS is known to exhibit developmental toxicity and postnatal effects, as has been demonstrated in experimental animal studies (Lau et al. 2003; Thibodeaux et al. 2003). In those studies, exposure to PFOS during pregnancy led to significant physiologic alterations that indicate maternal toxicity. In addition, these results indicate that *in utero* exposure to PFOS severely compromises postnatal survival of neonatal rats and mice and causes delays in growth and development accompanied by hypothyroxinemia in the surviving rat pups. However, little research has been conducted on the effects of PFOS on the human fetus, including epidemiologic, analytic, and toxicologic studies. It is necessary to investigate PFOS effects on thyroid hormone levels in a large number of fetuses. PFOS affects the estrous cycle and functions as an endocrine disruptor (Austin et al. 2003). Thyroid hormones play an important role in brain development, and deficiencies in T_4_ are known to cause mental delay in humans. In the present study, there was no apparent association between fetal PFOS concentration and thyroid hormones; however, the sample size may have been too small to detect such a relationship in a human population. Large-scale follow-up studies are necessary to assess the adverse effects of exposure to PFOS and related compounds on fetal development. Further exposure assessment studies of PFOS in the susceptible population during pregnancy are needed to determine whether maternal exposure to PFOS can lead to adverse effects on the endocrine system in offspring. The description of a large-scale fetal population and the effects of PFOS on the endocrine system will be detailed in a forthcoming paper.

## Figures and Tables

**Table 1:** Characteristics of mothers and infants (*n* = 15).

Characteristics	Median (range)
Maternal age (years)	28.4 (17–37)
Gestation (weeks)	39.7 (38–41)
Maternal height (cm)	157.2 (148–168)
Maternal prepregnancy weight (kg)	50.3 (40–61)
Maternal weight at delivery (kg)	60.4 (49.1–72)
Infants	
Male [*n* (%)]	8 (53.3)
Female [*n* (%)]	7 (46.7)
Birth weight (g)	3125.7 (2,579–4,162)

**Table 2:** Concentrations (ng/mL) of FOC congeners (PFOS, PFOA, and PFOSA) in maternal and cord blood samples (*n* = 15).

	Maternal			Fetal		
Sample no.	PFOS	PFOA	PFOSA	PFOS	PFOA	PFOSA
1	10.4	0.7	ND	3.9	ND	ND
2	17.6	2.3	ND	5.3	ND	ND
3	9.5	ND	ND	3.9	ND	ND
4	7.9	ND	ND	2.4	ND	ND
5	12.8	ND	ND	4.7	ND	ND
6	5.4	ND	ND	1.6	ND	ND
7	7.9	ND	ND	2.3	ND	ND
8	9.5	0.5	ND	3.0	ND	ND
9	7.9	ND	ND	2.4	ND	ND
10	5.8	ND	ND	2.1	ND	ND
11	9.9	ND	ND	2.8	ND	ND
12	8.1	ND	ND	2.5	ND	ND
13	4.9	ND	ND	1.7	ND	ND
14	8.3	ND	ND	2.8	ND	ND
15	6.9	ND	ND	1.6	ND	ND

ND, not detected: PFOS < 0.5 ng/mL; PFOA < 0.5 ng/mL; PFOSA < 1.0 ng/mL.
